# Editorial: Microorganisms for a Sustainable Viticulture and Winemaking

**DOI:** 10.3389/fmicb.2018.02650

**Published:** 2018-11-07

**Authors:** Gustavo Cordero-Bueso, Pedro Miguel Izquierdo-Cañas, Giovanna Suzzi

**Affiliations:** ^1^Department of Biomedicine, Biotechnology and Public Health, University of Cádiz, Cádiz, Spain; ^2^Instituto de la Vid y del Vino de Castilla la Mancha, Tomelloso, Ciudad Real, Spain; ^3^Faculty of Bioscience, University of Teramo, Teramo, Italy

**Keywords:** yeasts-like fungi, lactic acid bacteria, mixed fermentations, biotechnological and environmental applications, biodiversity

During the last decades, wine production in most countries is based on the use of commercial yeast and lactic acid bacteria strains leading to the colonization of the wineries and vineyards by these strains, and in the increasing use of chemical pesticides to control plant diseases or pests management. This signifies the consequent reduction of autochthonous microbial biodiversity. This signifies that wine styles could also become standardized, severely reducing the competitiveness of wines traditionally produced in the EU vs. the new wines elaborated in the emerging vine-growing areas. Moreover, the current climatic change, increasing of population, migration movements and economic changes need new strategies for viticulture and winemaking to ensure an economically or environmentally sustainable and healthy chain of production. Diversity and natural ecosystems could serve winemaking in many different ways, not all of which are well known. Thus, in this context increasing attention is being paid to species isolated from local vines or pristine environments and to their potential for stabilizing yields and reducing losses caused by plant diseases, pests and abiotic stresses and for safety and a better control of the fermentation processes using locally selected yeasts.

The research topic “Microorganisms for a sustainable viticulture and winemaking” belongs to the Food Microbiology section in the Frontiers in Microbiology journal. It covers a review and 14 original research papers. We present an overview of these papers starting with microbial populations associated with grape-berries and wines. Six of the contributions focused on the importance of the use of native microorganisms associated to grape-berries and uninoculated wines, as well as on the synergies and trade-offs that occur using selected native yeast strains as single or mixed starters. Vigentini et al. reported that in-bottle fermentation of sparkling wines is currently triggered by few commercialized *Saccharomyces cerevisiae* strains. This lack of diversity in *tirage* yeast cultures leads to a prevalent uniformity in sensory profiles of the final products. Authors exploited the natural multiplicity of yeast populations to introduce variability in sparkling wines throughout the re-fermentation step, considering it a convenient way for introducing differentiation to the final product without modifying the traditional technology. The work of Padilla et al. aimed at the reproduction of the native microbiota from the vineyard in the inoculum. Native selected *Saccharomyces* and non-*Saccharomyces* yeast species were inoculated sequentially into musts, and wines obtained were of similar quality and clearly differentiated by sensory analysis panelists. The fact that the proposed use of new starters using native yeast strains will almost invariably involve either simultaneous or sequential inoculation with *S. cerevisiae* has also driven the attention to the potential biological interactions between different starters during wine fermentation. Curiel et al. delved the response, under aerobic conditions, of *S. cerevisiae* to other two non-*Saccharomyces* species, *Hanseniaspora uvarum* and *Candida sake*, and focusing on the early stages of the interaction. Results point to some common features of the way *S. cerevisiae* modified its transcriptome in front of other yeast species, namely activation of glucose and nitrogen metabolism, being the later specific for aerobic conditions. Sadoudi et al. showed for the first time that the entire acetic acid and glycerol metabolic pathways can be modulated in *S. cerevisiae* by the presence of *Metschnikowia pulcherrima* at the beginning of fermentation. On the other hand, Lleixà et al. emphasized the importance of the concentration of nutrients on the evolution of mixed fermentations and points to the optimal conditions for a stable fermentation in which the inoculated yeasts survived until the end. According to García et al. choosing well the inoculation strategy between *S. cerevisiae* and non-*Saccharomyces* strains are critical to obtain a good quality wine. They analyzed, by real-time quantitative PCR (qPCR) combined with the use of specific primers, the dynamics of mixed and sequential cultures throughout the fermentation process at pilot scale using the Malvar white grape variety.

It is important to notice that the processes of yeast selection for using as wine fermentation starters have revealed a great phenotypic diversity both at the interspecific and intraspecific level, which is explained by a corresponding genetic variation among different yeast isolates. Guillamón and Barrio reviewed the mechanisms involved in generating genetic polymorphisms in yeasts, the molecular methods used to unveil genetic variation, and the utility of these polymorphisms to differentiate strains, populations, and species in order to infer the evolutionary history and the adaptive evolution of wine yeasts, and to identify their influence on their biotechnological and sensorial properties. Molecular tools have widely contributed to the interpretation of gene functionality within haploid isolates, but the genetics of metabolism in relevant polyploid yeast strains is still poorly understood. Vigentini et al. applied Clustered Regularly Interspaced Short Palindromic Repeats (CRISPR) and CRISPR-associated protein (Cas) system to two strains of *S. cerevisiae*, eliminating the *CAN1* arginine permease pathway to generate strains with reduced urea production. Moreover, other two original papers focused on models to predict the oenological potential of any given fermented beverage microbiome. In the first one, Bagheri et al. evaluated the complex wine microbiota by a model yeast consortium comprising eight species commonly encountered in South African grape musts and an ARISA based method to monitor their dynamics. The dynamics of these species were evaluated in synthetic must and Chenin blanc grape must fermentations in the presence or absence of *S. cerevisiae* using direct viable counts and ARISA. The data shown that *S. cerevisiae* specifically suppresses certain species while appearing to favor the persistence of other species. Thus, authors concluded that the wine ecosystem could be characterized by both mutually supportive and inhibitory species. Huang et al. proposed metatranscriptomics as a method to comprehensively explore the active microbial community members and key transcripts with significant functions in Chinese liquor starter production processes.

Native lactic acid bacteria (LAB) are also capable of growing during winemaking, thereby strongly affecting wine quality. Recently many winemakers are exploiting the potential of locally selected LAB strains able to be used as starters in detriment of commercial ones. The original research by Miranda-Castilleja et al. evidences the presence of local strains able to be used as starter cultures, and enable the assessment of the risks derived from the presence of spoilage LAB strains resistant to wine-like conditions. Other investigation carried out by Romero et al. revealed the presence of local strains distinguishable from commercial strains at the genetic/genomic level and having genomic traits that enforce their potential use as starter cultures in red wines.

With concern of safety and pest management, three original researches overview different prevention or correction strategies, using selected yeast, from the vineyard to the winery. The increasing level of hazardous residues in the environment and food chains has led the European Union to restrict the use of chemical fungicides. Thus, as stated before, exploiting new natural antagonistic microorganisms against fungal diseases could serve the agricultural production to reduce pre- and post-harvest losses, to boost safer practices for workers and to protect the consumers' health. Cordero-Bueso et al. evaluated the antagonistic potential of epiphytic yeasts against *Botrytis cinerea, Aspergillus carbonarius*, and *Penicillium expansum* pathogen species. In particular, yeast isolation was carried out from grape berries of *Vitis vinifera* ssp *sylvestris* populations, of the Eurasian area, and *V. vinifera* ssp *vinifera* cultivars from three different farming systems (organic, biodynamic, and conventional). They found six strains, all isolated from wild vines, with a notable antifungal action. On the other hand, it is well known that copper is widely used in agriculture as a traditional fungicide in organic farming to control downy mildew on grapes-berries, consequently it is possible to find this metal during all stages of the vinification process. Thus, Capece et al. found a wild yeast strain of *S. cerevisiae* able to complete the alcoholic fermentation and remove the copper from wine. This fact represents a biotechnological sustainable approach, as an alternative to the chemical-physical methods, ensuring at the same time a completed alcoholic fermentation and organoleptic quality of the wine. Furthermore, sulfur dioxide (SO_2_) is used commonly to stabilize the final product, but limiting its use is advised to preserve human health and boost sustainability in winemaking. Valdetara et al. investigated the influence of SO_2_ in relation with pH and ethanol factors on the expression of several genes and volatile phenol production in *Dekkera bruxellensis* under different model wines throughout a response surface methodology. The obtained results could be useful to improve the SO_2_ management at the grape harvesting and during winemaking to minimize the *D. bruxellensis* spoilage.

The varied contributions to this Research Topic are evidence of the study undertaken by researchers that embracing a sustainable agriculture would bring to the field of food microbiology, while warning that, at least for now, some selected microorganisms could replace agro-chemicals and standardized fermented beverages. Several of the issues surrounding new bioscience techniques, novel information about the selection of yeast starters, and alternatives to the use of some chemical compounds were also raised. We hope that research topic adequately informs readers about the benefits that nature offers to the field of food microbiology and about the many challenges that have yet to be overcome in this field.

## Author contributions

GC-B wrote and drafted the Editorial. PI-C and GS drafted and revised the final version of the Editorial.

### Conflict of interest statement

The authors declare that the research was conducted in the absence of any commercial or financial relationships that could be construed as a potential conflict of interest.

